# Multiple Intermolecular Interaction to Improve the Abrasion Resistance and Wet Skid Resistance of Eucommia Ulmoides Gum/Styrene Butadiene Rubber Composite

**DOI:** 10.3390/ma14185246

**Published:** 2021-09-12

**Authors:** Mingyang Li, Kuiye Wang, Yuzhu Xiong

**Affiliations:** Department of Polymer Materials and Engineering, College of Materials and Metallurgy, Guizhou University, Guiyang 550025, China; lmy897817576@126.com (M.L.); 15705427406@163.com (K.W.)

**Keywords:** silica, synergetic modification, compound material, wear resistance, wet skid resistance

## Abstract

A rubber composite was prepared by using methyltriethoxysilane (MTES) to modify silica (SiO_2_) and epoxidized eucommia ulmoides gum (EEUG) as rubber additives to endow silica with excellent dispersion and interfacial compatibility under the action of processing shear. The results showed that compared with the unmodified silica-reinforced rubber composite (SiO_2_/EUG/SBR), the bound rubber content of MTES-SiO_2_/EEUG/EUG/SBR was increased by 184%, and its tensile strength, modulus at 100% strain, modulus at 300% strain, and tear strength increased by 42.1%, 88.5%, 130.8%, and 39.9%, respectively. The Akron abrasion volume of the MTES-SiO_2_/EEUG/EUG/SBR composite decreased by 50.9%, and the wet friction coefficient increased by 43.2%. The wear resistance and wet skid resistance of the rubber composite were significantly improved.

## 1. Introduction

Silica is an important reinforcing material in industrial production [1]. In the 1940s, the production of silica was industrialized. Today, in addition to being used in food, toothpaste, ink, and pesticides, silica is also widely used in the rubber industry as a reinforcement [2,3]. In the tire industry, adding silica can reduce the heating and rolling resistance of the tread rubber [4,5], and improve the wet skid resistance and abrasion resistance of the tire [6,7,8,9,10]. Therefore, silica is a tire reinforcing filler with great development prospects [11,12,13,14,15]. However, the dispersibility of silica in the composite material is rather poor and the agglomeration is serious because of the large quantities of silanol groups on the surface of silica and high surface energy [16,17,18,19]. Therefore, improving the dispersion [20,21,22,23,24] of silica in composite materials by pretreatment is a hot research topic [25,26,27,28,29].

The silane coupling agent can react with silanol on the surface of silica to form covalent bonds, reduce the silanol density of the silica surface, and improve the compatibility of silica with the rubber matrix, leading to the improvement in filler dispersion and the performance of the rubber composite [30]. Bertora [31] used the small molecule coupling agent mercaptosilane (KH590) to graft liquid polybutadiene on the surface of silica and add it to the SBR, where the surface hydrophobicity of the rubber increased, the degree of filler aggregation decreased, and the dispersibility was improved. Dong [32] studied the effect of modified silica on the vulcanization kinetics of natural rubber (NR)/styrene butadiene rubber (SBR) blends. It was found that the vulcanization rates of NR and SBR phases in blends filled with KH560-modified silica were almost the same, showing a better co-vulcanization effect. In addition, compared with untreated silica, modified silica could be uniformly dispersed and improved the mechanical strength of the rubber blend [33,34].

The polar groups in polymers can interact with silica by forming hydrogen bonds or covalent bonds. Thus, polymers with polar groups have been used as alternatives to silane couplings [35,36]. Eucommia ulmoides gum (EUG) is a natural rubber whose main component is trans-polyisoprene, which is an isomer of natural rubber. Epoxidation is a popular chemical modification method for polydiolefin rubbers. The epoxidized eucommia ulmoides gum (EEUG) shows a higher polarity than original EUG because of the epoxy groups in its structure [37]. EEUG has been used as a compatibilizer in filled systems. Wang [38] modified silica with a macromolecular modifier (EEUG) and added it to SBR. The dispersion of modified silica was better, the wear resistance of the compound was improved, and the wear volume decreased from 0.192 cm^3^ to 0.179 cm^3^. 

**Figure 1:**
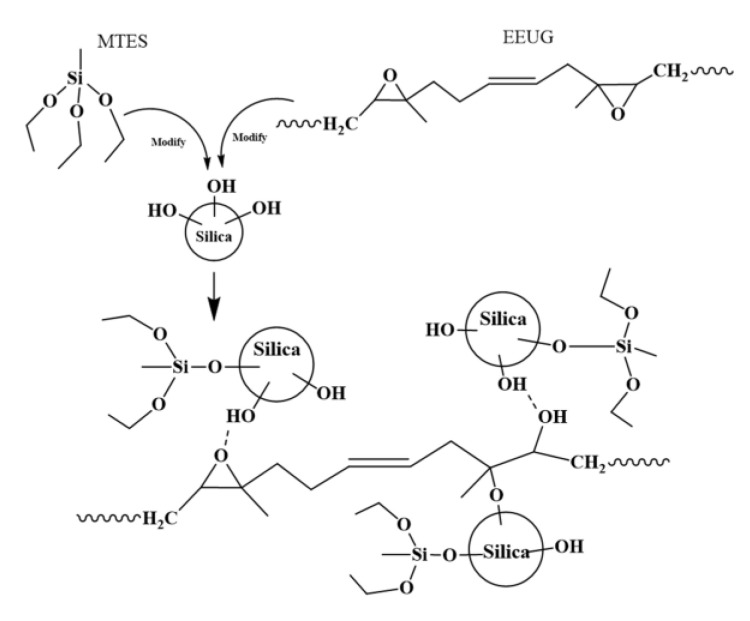
Schematic diagram of the synergistic effect of methyltriethoxysilane (MTES) and epoxidized eucommia ulmoides gum (EEUG) on silica.

In this work, we first modified the silica with a small molecule coupling agent methyltriethoxysilane (MTES), and added it to the rubber matrix together with EEUG to prepare the composite material. The schematic diagram of the synergistic effect of MTES and EEUG on silica is presented in Figure 1. The small-molecule coupling agent (MTES) reacted with the silanol groups on the surface of SiO_2_ to form a covalent bond, which reduced the surface activity of SiO_2_ and reduced its agglomeration tendency. The epoxy group on the EEUG macromolecule can form hydrogen bonds or undergo a ring-opening reaction with the silanol groups on the surface of silica to form a covalent bond; this anchors part of the silica to the EEUG molecular chain, which increases the silica and interfacial compatibility of the rubber matrix. During processing, the shear flow of EEUG molecular chains drives better dispersion of silica to obtain rubber composites with excellent properties.

## 2. Materials and Methods

### 2.1. Materials

Eucommia ulmoides gum (EUG) was purchased from Shandong Qingzhou Beilong Company (Weifang, China); SBR was purchased from Lanzhou Petrochemical Co. Ltd. (Lanzhou, China); silica (SiO_2_) was purchased from Changzhou Lehuan Chemical Co. Ltd. (Changzhou, China); methyltriethoxysilane (MTES), sodium dodecylbenzene sulfonate, hydrogen peroxide (H_2_O_2_), and sodium bicarbonate (NaHCO_3_) were purchased from Shanghai Aladdin Biochemical Technology Co. Ltd. (Shanghai, China); zinc oxide (ZnO), stearic acid (SA), accelerator (DM), antioxidant 4020, and sulfur (S) were obtained from Guizhou Tire Factory (China). Petroleum ether and absolute ethanol were purchased from Chongqing Chuandong Chemical Co. Ltd. (Chongqing, China). Formic acid (HCOOH) was purchased from Tianjin Fuyu Fine Chinese Industrial Co. Ltd. (Tianjin, China). Deionized water was made in-house.

### 2.2. Preparation of EEUG

EUG (50 g) was added to a 2000 mL beaker, followed by the addition of 900 mL petroleum ether. Then, the beaker was placed in a 45 °C constant-temperature water bath, and mechanically stirred until the gum was completely dissolved. Second, 300 mL deionized water and 2.5 g sodium dodecylbenzene sulfonate were added, and stirring was continued to form a stable emulsion. Then, quantitative HCOOH was added, and the corresponding amount of H_2_O_2_ was slowly added (the molar ratio of double bonds, HCOOH, and H_2_O_2_ was 1:0.8:2). The reaction lasted for 1 h at 45 °C under mechanical stirring. After epoxidation, hot saturated NaHCO_3_ solution was added to the beaker to adjust the pH of the EEUG solution to 7. Then, absolute ethanol was added to precipitate EEUG. The product was dried to constant weight at room temperature to obtain 49.25 g of the desired EEUG. A small amount of EEUG was mixed with unmodified SiO_2_ (denoted as EEUG-SiO_2_) for infrared spectroscopy (Nexus 6700, Thermo Scientific, MA, USA) and XPS spectrum analysis (Thermo Fisher K-Alpha, Shenzhen Junhuiteng Technology Co. Ltd., Shenzhen, China).

### 2.3. MTES Modified SiO_2_

A tray of SiO_2_ was placed in a vacuum oven at 80 °C for 12 h to remove moisture. Dried SiO_2_ (30 g) was poured into a three-necked flask, and then 450 mL absolute ethanol was added, with ultrasonic treatment for 35 min. MTES (6 g) was poured into a beaker, and then 60 mL absolute ethanol was added, and ultrasonic treatment was performed to form an emulsion. A three-necked flask containing SiO_2_ was placed in a 60 °C water bath, mechanically stirred, and heated at reflux. The pre-emulsified MTES was then added dropwise. The product was taken out after 2 h, aged for 1 h, and washed three times with absolute ethanol. After suction filtration, it was dried in a vacuum oven at 80 °C to obtain MTES-modified SiO_2_ (denoted as MTES-SiO_2_). A small amount of EEUG was mixed with MTES-SiO_2_ (denoted as EEUG-MTES-SiO_2_) for infrared spectroscopy and XPS spectrum analysis.

### 2.4. Preparation of Rubber Composites

According to the ratios listed in Table 1, SBR, EUG, EEUG, SiO_2_, and MTES-SiO_2_ were mixed in three stages in an internal mixer (XSM-05, Shanghai Kechuang Rubber Machinery Equipment Co. Ltd., Shanghai, China), each with a temperature of 125 °C, 130 °C, and 135 °C, a rotor speed of 80 rpm, and a mixing time of 7 min. After the mixture was cooled, the roller temperature of the open mill (Φ160 × 320, Dongguan Changfeng Rubber Machinery Co. Ltd., Dongguan, China) was adjusted to about 70 °C. The prepared mixture was placed on the open mill for 3 min and passed twice. It was used to wrap the roller, and then ZnO, SA, antioxidant 4020, DM, and S were added sequentially according to the ratio in Table 1. After all the ingredients were added, it was thinned up to five to seven times, and then the roller distance was evenly and slowly adjusted to the left and right, so that the mixture was discharged with a thickness of about two millimeters. The obtained sheet-like mixture was laid flat at room temperature and a humidity of 40–50% for 24 h to eliminate internal stress. Then, a part of the mixture was used to make samples by air pressure by putting round cake-shaped test samples in a rotorless auto-vulcanization instrument (MD-3000A, Taiwan High-Speed Rail Technology Co. Ltd., Taichung City, Taiwan) to determine the positive vulcanization time. Then, the mixture was cut into a size suitable for the vulcanization mold. It was put into a flat vulcanizer (XLB-25t, Jiangdu Mingzhu Experimental Machinery Factory, Yangzhou, China) at a vulcanization temperature of 150 °C. The vulcanization time was based on the positive vulcanization time obtained by the test. The prepared rubber composite material was laid flat at room temperature and a humidity of 40–50% for 24 h to eliminate internal stress.

The formulation of the rubber composite is shown in Table 1, where #1 is a composite prepared from unmodified SiO_2_, EUG, and SBR, denoted as SiO_2_/EUG/SBR; #2 is a composite prepared from MEST-SiO_2_, EUG, and SBR, denoted as MEST-SiO_2_/ EUG/SBR; #3 is a composite prepared from unmodified SiO_2_, EEUG, EUG, and SBR, denoted as SiO_2_/EEUG/EUG/SBR; and #4 is a composite prepared from MTES-SiO_2_, EEUG, EUG, and SBR, denoted as MTES-SiO_2_/EEUG/EUG/SBR.

### 2.5. Testing and Characterization

#### 2.5.1. Fourier-Transform Infrared (FTIR) Spectroscopy

Unmodified SiO_2_, MTES, EEUG, EEUG-SiO_2_, MTES-SiO_2_, and EEUG-MTES-SiO_2_ were characterized with a Fourier-transform infrared spectrometer (Nexus 6700, Thermo Scientific, MA, USA), with a scanning wavenumber range of 400–4000 cm^−1^.

#### 2.5.2. X-ray Photoelectron Spectroscopy (XPS)

XPS spectra of SiO_2_, MTES-SiO_2_, EEUG-SiO_2_, and EEUG-MTES-SiO_2_ were recorded by using an X-ray photoelectron spectrometer (Thermo Fisher K-Alpha, Shenzhen Junhuiteng Technology Co. Ltd., Shenzhen, China). Samples were analyzed under vacuum (*p* < 10^−8^ mbar) with a pass energy of 150 eV (survey scans) or 50 eV (high-resolution scans). All peaks were calibrated with C1s peak binding energy at 284.8 eV for adventitious carbon.

#### 2.5.3. Determination of Bonding Rubber

Rubber (0.5 g) was crushed with scissors into small pellets, wrapped in copper mesh, and immersed in 60 mL of toluene for three days. The toluene was refreshed every 24 h. After that, it was immersed in 600 mL of acetone for 24 h. The toluene was removed, and the remaining rubber was placed in an oven at 60 °C for drying until its mass did not change; each sample was tested for three times. The bonding rubber content w was calculated as follows:

(1) $$w=\frac{w_{1}-(w_{2}-w_{3})}{w_{1}}\times 100\%$$

where *w*_1_ is the rubber mass of the sample; *w*_2_ is the mass of rubber and copper mesh; and *w*_3_ is the mass of the remaining rubber and copper mesh after drying to a constant weight.

#### 2.5.4. Curing Characteristics 

A rotorless vulcanizer (MD-3000A, Taiwan High-Speed Rail Technology Co. Ltd.) was used to test the curing characteristics of the rubber composite. The test temperature was 150 °C, the test time was 40 min, the rotation angle was 0.5°, the stabilization time was 1 s, and the stability range was 0.50 °C; each sample was tested twice.2.5.5. Rubber Processing Analyzer 

A rubber processing analyzer (RPA2000, Alpha, Technologies, Hudson, OH, USA) was used to determine the storage modulus *G′* and loss factor *tanδ* of the rubber composite, with a strain sweep range of 0.7–400%, temperature of 60 °C, and frequency of 1 Hz.

#### 2.5.5. Mechanical Performance Testing

According to GB/T528-1998, the rubber was prepared into dumbbell-shaped specimens and analyzed with a universal material testing machine (Inspeakt Table 10 kN, Germany Huibo Material Testing Company, Beijing, China). The tensile rate was 500 mm/min, and each sample was tested five times.

#### 2.5.6. Akron Abrasion Test

A special mold was used to prepare the rubber material into a strip sample, which was then glued to a rubber wheel, and allowed to stand for 8 h. Then, a testing instrument (ZB-201, Jiangsu Zhengrui Taibang Electronic Technology Co. Ltd., Yangzhou, China) was used to test the Akron abrasion volume (*V*) using a test angle of 15°. First, the sample was pre-ground 600 revolutions, and the fallen rubber crumbs were collected and weighed, accurate to 0.001 g, and the weight was recorded as *m*_1_. The pre-ground sample was ground for another 3416 revolutions, then the fallen rubber crumbs were collected and weighed again, and the weight was recorded as *m*_2_. The density (*ρ*) of the sample was tested according to GB/T533; each sample was tested three times. The Akron abrasion volume *V* was calculated using the following equation:

(2) $$V=\frac{m_{1}-m_{2}}{\rho }$$

#### 2.5.7. SEM Analysis 

Scanning electron microscope (SEM, JSM-7500F, JEOL Ltd., Beijing, China) was used to observe the tensile fracture surface morphology of the composite and the wear surface morphology after Akron abrasion tests.

#### 2.5.8. Wet Sliding Friction Test

A pendulum friction coefficient tester (BM-III) was used to test the friction coefficient of the rubber composite under wet and slippery conditions. The sliding path was 126 mm, the test temperature of the rubber compound was 25 °C, and the humidity was between 45–55%. Water was sprayed on the glass surface to simulate a wet road. Five rubber sliders were prepared for each composite, and water was sprayed on the glass surface again before each test [9].

## 3. Results

### 3.1. FTIR Analysis of the Interaction between SiO_2_ and Enhancer

**Figure 2:**
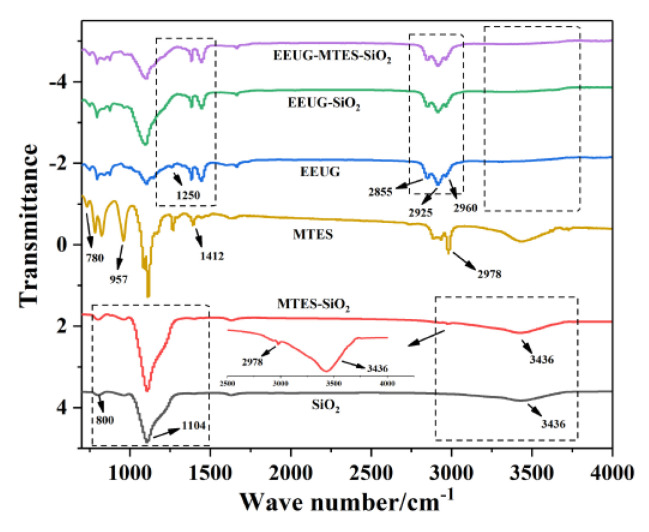
FTIR spectra of EEUG, MTES, SiO_2_, and modified SiO_2_.

Figure 2 shows the FTIR spectra of unmodified SiO_2_, MTES-SiO_2_, EEUG-SiO_2_, EEUG-MTES-SiO_2_, and EEUG. The FTIR spectrum of unmodified SiO_2_ contained peaks for the asymmetric stretching vibration of Si–OH at 3436 cm^−1^, the asymmetric stretching vibration of Si–O–Si and the symmetric stretching vibration peak of Si–O at 1104 cm^−1^ and 800 cm^−1^ [5,36]. In the infrared spectrum of MTES, the peak at 780 cm^−1^ assigns to the bending vibration of Si–C, the peaks at 957 cm^−1^, 1412 cm^−1^, and 2978 cm^−1^ corresponded to the characteristic peak of Si–O–CH_2_–, the absorption peak of C–H in methyl group, and the stretching vibration peak of –CH_3_ [39,40]. In the infrared spectrum of EEUG, the peaks at 2963 cm^−1^, 2925 cm^−1^, and 2855 cm^−1^ corresponded to the asymmetric stretching vibration of methyl groups, asymmetric stretching vibration, and symmetric stretching vibration of methylene groups, respectively. The peak at 1250 cm^−1^ is the symmetrical stretching vibration of epoxy group [36]. Comparing the spectra of SiO_2_ and MTES-SiO_2_, a new stretching vibration peak of -CH_3_ appeared at 2978 cm^−1^ [39], indicating that MTES was successfully grafted onto SiO_2_. In the spectra of unmodified SiO_2_, EEUG, and EEUG-SiO_2_, it can be seen that compared with unmodified SiO_2_, the EEUG-SiO_2_ spectrum contained three characteristic peaks belonging to EEUG at 2963 cm^−1^, 2925 cm^−1^, and 2855 cm^−1^. Compared with the infrared spectrum of EEUG, the epoxy group peak at 1250 cm^−1^ disappeared [37] and the Si–OH peak at 3436 cm^−1^ weakened in the EEUG-SiO_2_ spectrum, indicating that the epoxy group of EEUG was opened by the hydroxyl group on the SiO_2_ surface. The infrared spectrum of MTES-EEUG-SiO_2_ was roughly the same as that of EEUG-SiO_2_ because the -CH_3_ absorption peak on MTES was incorporated into the -CH_3_ absorption peak of EEUG. The above phenomena showed that both the individual modification and synergistic modification of SiO_2_ by the small-molecule coupling agent and the macromolecular modifier were successful.

### 3.2. XPS Analysis of the Interaction between SiO_2_ and Enhancer

**Figure 3:**
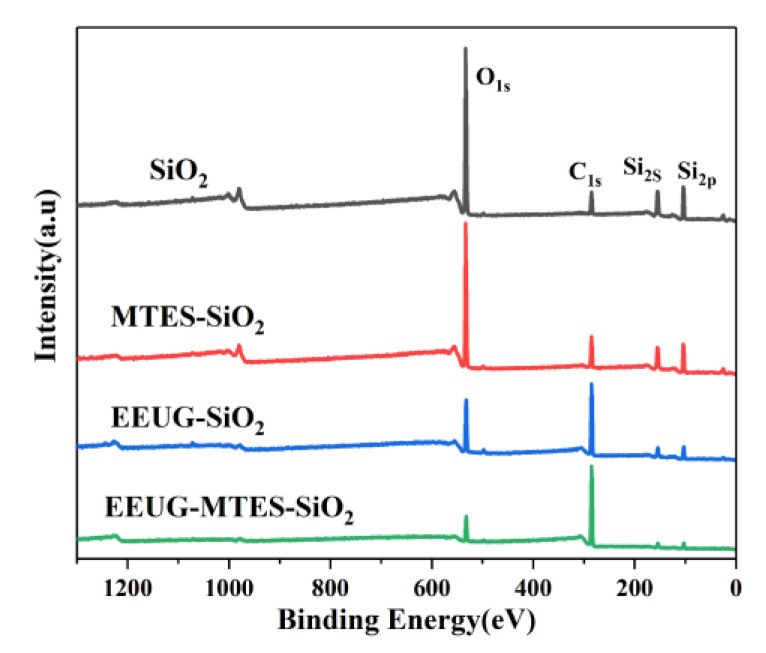
XPS wide-scan spectra of SiO_2_, MTES-SiO_2_, EEUG-SiO_2_, and EEUG-MTES-SiO_2_.

Figure 3 shows the XPS wide-scan spectra of SiO_2_, MTES-SiO_2_, EEUG-SiO_2_, and EEUG-MTES-SiO_2_ and the information of the elemental composition on the surface of the particle are shown in Table 2. It shows that the C1s intensity and element content of C increased and elemental content of Si, O decreased in MTES-SiO_2_, EEUG-SiO_2_, and EEUG-MTES-SiO_2_ compared to SiO_2_. This is due to that the reaction of EEUG and MTES on silica’s surfaces that results in the reduction of O and Si atoms and the increase in C atoms on the surfaces of the silica particles [41].

**Figure 4:**
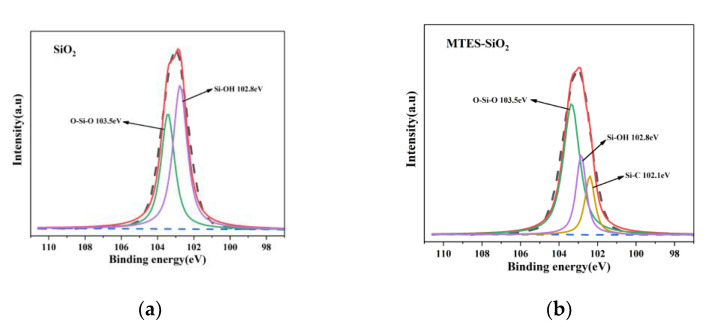

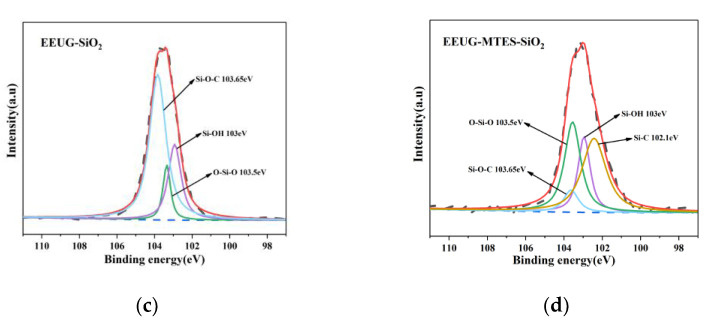
High-resolution XPS spectra of Si2p: (**a**) SiO_2_; (**b**) MTES-SiO_2_; (**c**) EEUG-SiO_2_; (**d**) EEUG-MTES-SiO_2_.

High-resolution XPS spectra of Si2p for SiO_2_, MTES-SiO_2_, EEUG-SiO_2_, and EEUG-MTES-SiO_2_ are shown in Figure 4. It can be seen that the binding energies for Si2p of O–Si–O and Si–O–H groups in SiO_2_ were 103.5 eV and 102.8 eV [36], respectively. In MTES-SiO_2_, a new peak appeared at 102.1 eV, which stands for the binding energy of Si2p in the Si-C group [42], indicating that MTES is grafted onto SiO_2_. In EEUG-SiO_2_ and EEUG-MTES-SiO_2_, the new peak at 103.65eV is caused by the Si–O–C group [36], implying that the ring opening reaction of epoxy group has taken place. Simultaneously, the binding energy for Si2p of Si–OH shifted to 103eV. This is because a hydrogen bond can form between the epoxy group and silanol, which influences the chemical environment around the Si atom, inducing the shifts of binding energy for Si2p of Si–OH [43,44].

### 3.3. Microstructure of Rubber Composite

**Figure 5:**
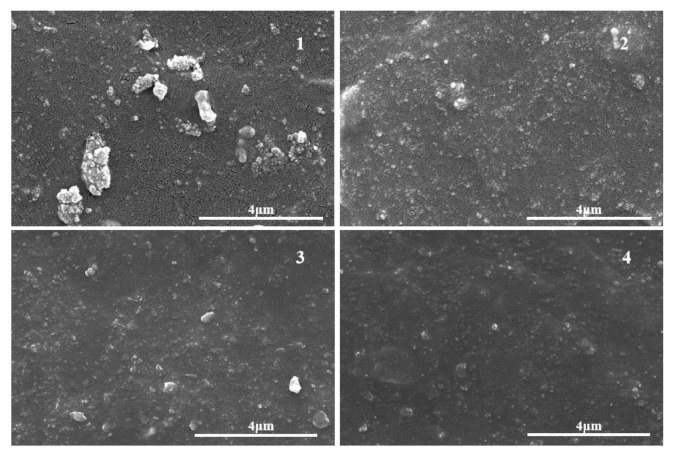
SEM image of the tensile fracture surface of the rubber composite: (1) SiO_2_/EUG/SBR; (2) MEST-SiO_2_/EUG/SBR; (3) SiO_2_/EEUG/EUG/SBR; (4) MTES-SiO_2_/EEUG/EUG/SBR.

Figure 5 shows an SEM image of the tensile fracture surface of the rubber composite. The SEM of SiO_2_/EUG/SBR showed that unmodified SiO_2_ formed poorly-dispersed large agglomerates in the rubber compound. Obvious phase separation occurred between SiO_2_ and the rubber matrix, and the interfacial adhesion between the two was poor [45]. The SEM image of MTES-SiO_2_/EUG/SBR showed that after SiO_2_ was modified by MTES, MTES covalently bonded with the silanol groups on SiO_2_. Therefore, the steric hindrance effect on the surface of SiO_2_ was improved, and the agglomeration of SiO_2_ was reduced [46], thus, the particle size of SiO_2_ dispersed in the material matrix was reduced and the dispersion was significantly improved. The SEM image of SiO_2_/EEUG/EUG/SBR showed that adding EEUG to the matrix also significantly improved the dispersion of SiO_2_. The SEM image of MTES-SiO_2_/EEUG/EUG/SBR showed that the synergy of MTES and EEUG improved the dispersion and compatibility of SiO_2_ with the matrix. The small-molecule coupling agent MTES weakened the degree of SiO_2_ aggregation, and the macromolecular modifier EEUG formed hydrogen bonds or bonded with silanol groups. At the same time, SiO_2_ bonded to the EEUG molecular chain was better dispersed in the matrix when the rubber molecular chains became entangled and flowed [36,47].

### 3.4. Binder Content of Rubber Composite

**Figure 6:**
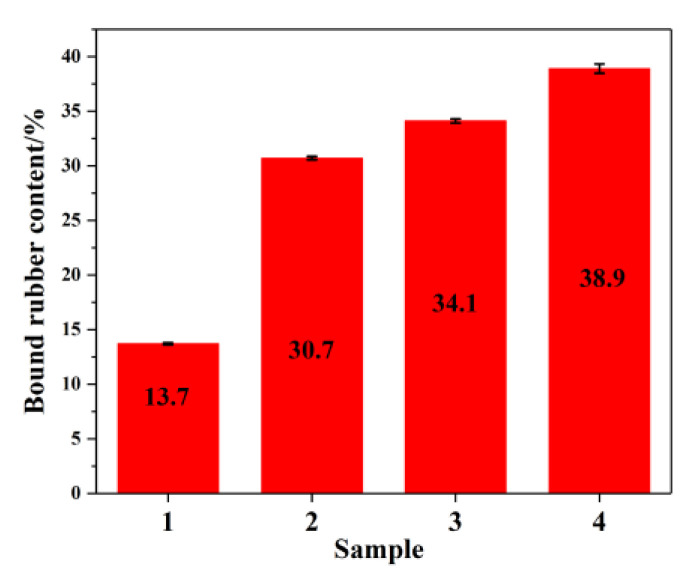
The binder content of rubber composites: (1) SiO_2_/EUG/SBR; (2) MEST-SiO_2_/EUG/SBR; (3) SiO_2_/EEUG/EUG/SBR; (4) MTES-SiO_2_/EEUG/EUG/SBR.

Figure 6 shows the binder content of rubber composites. Both MTES-modified SiO_2_ and the addition of EEUG increased the bound rubber content of the composite. Compared with unmodified SiO_2_, the bound rubber content of the composite with both EEUG and MTES-SiO_2_ added increased by 184%. Analysis showed that in the rubber composite materials, well-dispersed SiO_2_ with interfacial compatibility can increase the degree of interfacial interactions with the rubber matrix; thus, more molecular chains of the rubber matrix will be physically adsorbed on the surface of SiO_2_ [48,49]. At the same time, the EEUG molecular chains bonded with SiO_2_ will also become physically entangled with the rubber matrix [50], which greatly increases the material’s bound rubber content.

### 3.5. Curing Characteristics of Rubber Composites

**Figure 7:**
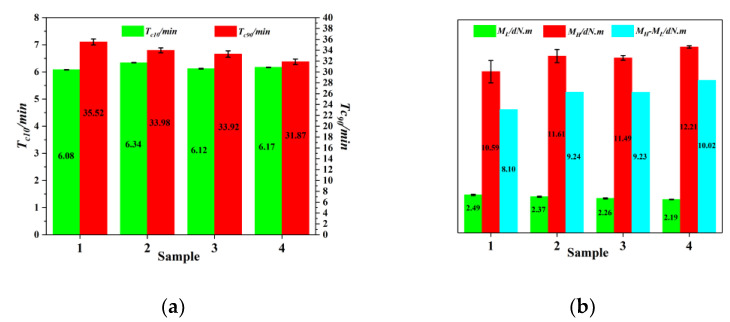
The curing characteristics of rubber: (**a**) The scorch time (*T_c10_*) and curing time (*T_c90_*); (**b**) The minimum torque (*M_L_*), maximum torque (*M_H_*) and torque difference (*M_H_-M_L_*); (1) SiO_2_/EUG/SBR; (2) MEST-SiO_2_/EUG/SBR; (3) SiO_2_/EEUG/EUG/SBR; (4) MTES-SiO_2_/EEUG/EUG/SBR.

The curing characteristics of rubber are important for the manufacture of rubber products. Figure 7 shows the curing characteristics of the rubber composites. Rubber composites should have suitable scorch time (*T_c10_*) and optimal curing time (*T_c90_*) to meet processing performance requirements [51]. The minimum torque (*M_L_*) characterizes the degree of interaction between filler particles. The smaller the value, the weaker the interactions between the filler, and the better the dispersion of the filler in the matrix [45]. The maximum torque (*M_H_*) reflects the degree of interactions between the filler and matrix [52], and the torque difference (*M_H_-M_L_*) value is positively correlated with the extent of crosslinking of the rubber compound [45]. It can be seen from Figure 7a that the addition of EEUG caused the *T_c10_* of the rubber compound to fluctuate slightly. This is because the internal epoxy group of the EEUG in the rubber compound opened and crosslinked under the action of heat or a coupling agent, thereby affecting the *T_c10_* [53,54]. Both MTES-modified SiO_2_ and the addition of EEUG reduced the *T_c90_* and *M_L_* of the compound, while the maximum torque (*M_H_*) and the torque difference (*M_H_-M_L_*) increased. This shows that the modified SiO_2_ had better dispersion and a higher degree of interaction with the matrix, and its composite had a higher crosslinking density. Analysis suggests that both the small-molecule coupling agent MTES and the macromolecular modifier EEUG bonded with the silanol groups on the surface of SiO_2_, thereby reducing the number of silanol groups on its surface. This decreased its surface polarity, weakened its agglomeration tendency, enhanced the dispersion of SiO_2_, and further reduced its adsorption of vulcanization accelerators, thereby reducing the vulcanization time [51,55,56]. Modified SiO_2_ had a weaker aggregation tendency, could be better dispersed in the rubber matrix, had a higher degree of interaction with the rubber matrix, and increased the crosslinking density.

### 3.6. Rubber Processing Analyzer of Rubber Composites

**Figure 8:**
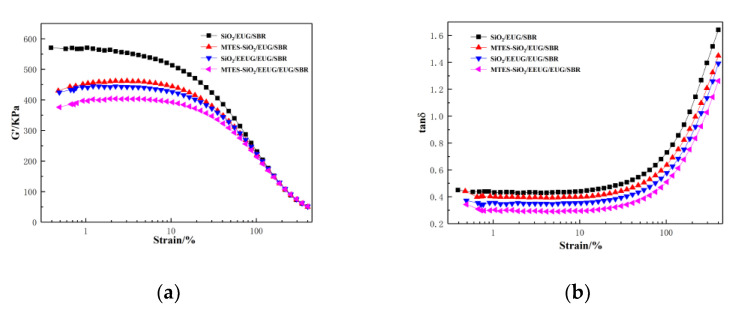
Rubber processing analyzer (RPA) curves of rubber composites: (**a**) *G′*–strain curve; (**b**) *tanδ*–strain curve.

The RPA results of rubber composites are shown in Figure 8. Generally, the storage modulus (*G′*) is used to indicate the degree of interaction between fillers inside rubber compounds. The phenomenon that *G′* decreases with the increase in strain is called the Payne effect [35]. When *G′* is large, the fillers strongly interact with each other, and the Payne effect is more obvious. It can be seen from Figure 8a that compared with the composite with unmodified SiO_2_, both MTES-modified SiO_2_ and the addition of EEUG significantly reduced the *G′* of the composite at low strain, while there was not a significant difference in the high-strain region. The composite with both EEUG and MTES-SiO_2_ added displayed the best filler dispersion, and its *G′* at low strain was the smallest, indicating the least interactions between fillers. This is because MTES reacts with the silanol groups on the surface of SiO_2_, reducing interactions between SiO_2_. At the same time, EEUG forms hydrogen bonds or covalent bonds with the silanol groups on the surface of SiO_2_, which further reduces the interactions between SiO_2_ [57]. It can be seen from Figure 8b that the loss factor (*tanδ*) of MTES-modified SiO_2_ and EEUG-added composites were both significantly lower at low strain compared with the composites with unmodified SiO_2_. The synergy of EEUG and MTES makes SiO_2_ produce the best dispersion and compatibility with the rubber matrix. Well-dispersed fillers further limit the mobility of the matrix molecular segments, and the abrasion between fillers and the internal friction loss of the rubber matrix molecular segments can be reduced [8,38]. Therefore, the MTES-SiO_2_/EEUG/EUG/SBR composite had the lowest loss factor.

### 3.7. Mechanical Performance of Rubber Composites

**Figure 9:**
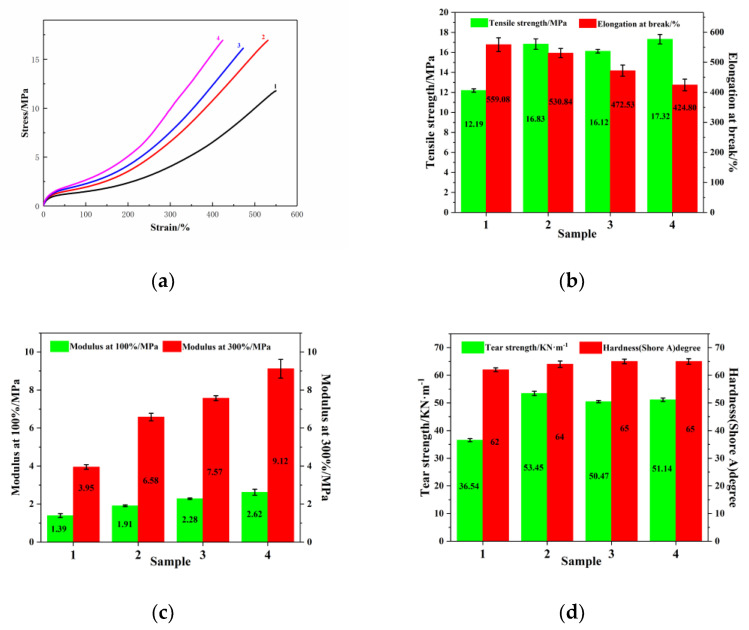
(**a**) The stress–strain curves of rubber composites. (**b**–**d**) The mechanical properties of the rubber composites. (1) SiO_2_/EUG/SBR, (2) MEST-SiO_2_/EUG/SBR, (3) SiO_2_/EEUG/EUG/SBR, (4) MTES-SiO_2_/EEUG/EUG/SBR.

Figure 9a shows the stress–strain curves of the rubber composites, and Figure 9b–d shows the mechanical properties of the rubber composites. It can be seen from Figure 9a that both MTES-modified SiO_2_ and the addition of EEUG increased the modulus of the composite. The composite with both EEUG and MTES-SiO_2_ added had the highest modulus, which was attributed to the good dispersion of SiO_2_ in the matrix. It can be seen from Figure 9b–d that both MTES-modified SiO_2_ and the addition of EEUG improved the tensile strength, modulus at 100% strain, modulus at 300% strain, and tear strength of the rubber composite. The Shore hardness of the rubber composite material was basically unchanged, while still maintaining good elongation at break. The good dispersion of SiO_2_ and its compatibility with the matrix endowed the composite with excellent comprehensive properties. Compared with the composite with unmodified SiO_2_, the tensile strength of the composite with both EEUG and MTES-SiO_2_ added increased by 42.1%, the modulus at 100% strain increased by 88.5%, the modulus at 300% strain increased by 130.8%, and the tear strength increased by 39.9%.

### 3.8. Wear Resistance and Wet Skid Resistance of Rubber Composites

**Figure 10:**
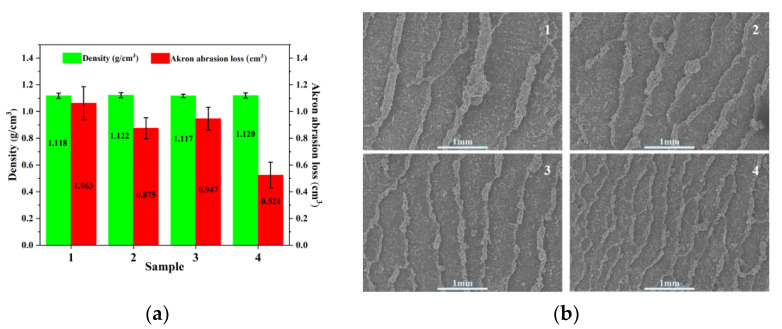
(**a**) The Akron abrasion and density of rubber composites; (**b**) SEM of the wear surface morphology of the rubber composite. (1) SiO_2_/EUG/SBR; (2) MEST-SiO_2_/EUG/SBR; (3) SiO_2_/EEUG/EUG/SBR; (4) MTES-SiO_2_/EEUG/EUG/SBR.

Figure 10a shows the Akron abrasion and density of rubber composites. The MTES-modified SiO_2_ and the addition of EEUG had little effect on the density of the composite, but it significantly reduced the Akron wear of the material and improved the wear resistance. Compared with the composite with unmodified SiO_2_, the abrasion volume of the composite with both EEUG and MTES-SiO_2_ added was reduced by 50.9%, and the wear resistance was significantly improved. According to the analysis, in the compound with unmodified SiO_2_, the compatibility of the filler and the matrix was not good, and the large agglomerated particles easily separated from the matrix. The resulting cavity became a weak point of the compound. Upon further deformation of the rubber, these weak points formed cracks on the surface or inside the rubber, causing the wear volume to increase [58,59]. The synergy of EEUG and MTES allows SiO_2_ to be well-dispersed and compatible with the matrix. This limited the movement of the material’s molecular chain when external forces were applied, and improved the material’s ability to resist friction. At the same time, the good compatibility of SiO_2_ and the matrix increased the bonding strength between the filler and the matrix. This prevented it from being easily damaged during the friction process, so the abrasion volume was reduced [54]. Moreover, the better the dispersion of SiO_2_ particles, the more difficult the path of crack propagation, the smoother the path of stress transmission to the SiO_2_ particles, and the less likely the composite will form cracks on its surface [59,60]. This effectively improved the wear resistance of the composite.

Figure 10b shows a SEM image of the wear surface morphology of the rubber composite. The composite with unmodified SiO_2_ had high and wide ridges on its wear surface, and the distance between the ridges was relatively large. The ridges produced on the surface of the MTES-modified SiO_2_ and EEUG-added composite became shorter and narrower, and the distance between the ridges became smaller. This indicates that the wear resistance of the material was improved. The composite with both EEUG and MTES-SiO_2_ added had the shortest and narrowest ridges, and the distance between ridges was the smallest, indicating that it had the strongest resistance to friction and the best wear resistance.

**Figure 11:**
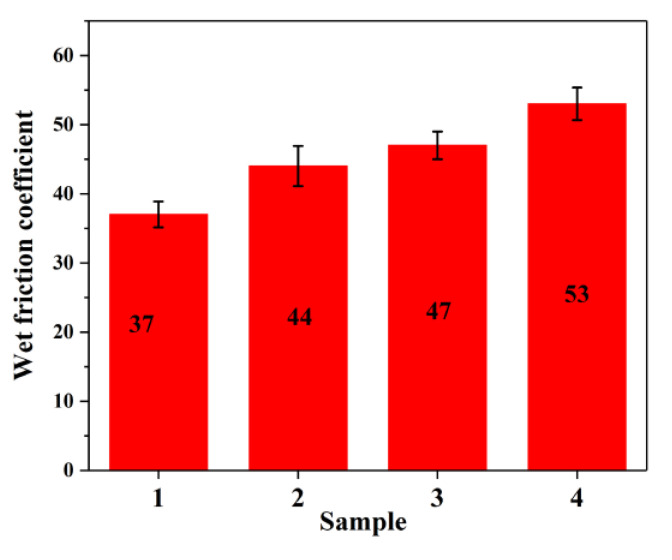
The wet friction coefficient of rubber composites. (1) SiO_2_/EUG/SBR; (2) MEST-SiO_2_/EUG/SBR; (3) SiO_2_/EEUG/EUG/SBR; (4) MTES-SiO_2_/EEUG/EUG/SBR.

Figure 11 shows the wet friction coefficient of the rubber composites. The MTES-modified SiO_2_ and the addition of EEUG increased the wet friction coefficient of the composite material. Compared with the composite with unmodified SiO_2_, the wet friction coefficient of the composite with both EEUG and MTES-SiO_2_ added increased by 43.2%. Analysis showed that SiO_2_ helped pierce the water film on the rubber surface and reduced the thickness of the water film, so the rubber could contact the ground faster, increased the contact area between the rubber and the ground, and achieved an anti-skid effect [9]. The dispersion of SiO_2_ in the MTES-SiO_2_/EEUG/EUG/SBR composite and its compatibility with the matrix were the best, which prevented it from separating from the matrix, the wet friction coefficient of the rubber composite was significantly improved, thereby improving the wet skid resistance of the rubber compound. This can help shorten the braking distance on wet roads and improve the safety factor of tires.

## 4. Conclusions

The synergistic effect of a small molecule coupling agent and macromolecular modifier significantly improved the dispersion of SiO_2_ in the rubber matrix and the interfacial compatibility with the matrix. The MTES reacted with the silanol groups on the surface of SiO_2_ to form a covalent bond, which reduced the surface activity and agglomeration tendency of SiO_2_. The epoxy group on the EEUG can form hydrogen bonds or covalent bonds with the silanol groups on the surface of SiO_2_, which anchors part of the SiO_2_ to the EEUG molecular chain. During processing, the shear flow of EEUG molecular chains can drive better dispersion of SiO_2_ in the rubber matrix. The results verified that compared to the composite with added unmodified SiO_2_, the composite with both EEUG and MTES-SiO_2_ added had higher binder content, better filler dispersion, better wear resistance, and wet skid resistance. Thus, this work exhibited great potential in the rubber and tire industry.

## Figures and Tables

**Table 1:** Rubber composite formula (phr).

Sample	#1	#2	#3	#4
SBR	70	70	70	70
EUG	30	30	24	24
EEUG	0	0	6	6
SiO_2_	30	0	30	0
MTES-SiO_2_	0	30	0	30
ZnO	5	5	5	5
SA	4	4	4	4
DM	2	2	2	2
Antioxidant 4020	1	1	1	1
S	2.5	2.5	2.5	2.5

**Table 2:** Comparison of atomic content (at.%) in the XPS spectra of SiO_2_, MTES-SiO_2_, EEUG-SiO_2_, and EEUG-MTES-SiO_2_.

Sample	Element Constitution (%)		
	Si	C	O
SiO_2_	26.91	18.85	54.24
MTES-SiO_2_	24.73	26.51	48.75
EEUG-SiO_2_	11.13	66.23	22.64
EEUG-MTES-SiO_2_	6.05	82.41	11.54

## Data Availability

Data are contained within this article.
